# Complete mitochondrial genome of *Sinibotia superciliaris* (Teleostei: Cypriniformes, Cobitidae) and its phylogenetic position

**DOI:** 10.1080/23802359.2019.1703596

**Published:** 2019-12-18

**Authors:** Chuntao Li, Zili Jiang, Qingrong Wang, Peiyong Song, Kaiqin Chen

**Affiliations:** College of Biology and Agriculture, Zunyi Normal College, Zunyi, China

**Keywords:** Mitogenome, *Sinibotia superciliaris*, Cobitidae, phylogeny

## Abstract

The complete mitochondrial genome of *Sinibotia superciliaris* was determined in this study. It contained 13 protein-coding genes (PCGs), 22 tRNA, 2 rRNAs, and a control region with the base composition 31.57% A, 27.18% C, 25.52% T, and 15.74% G. Here we compared this newly determined mitogenome with another one from the same species reported before. The variable sites and the genetic distances between the two mitogenomes were 20 bp and 0.1%. 15 variable sites were occurred in the PCGs. The results from the phylogenetic analysis showed that the genus *Sinibotia* is a monophyletic group and *S. superciliaris* demonstrate a sister relationship with *Sinibotia pulchra*.

*Sinibotia superciliaris* (Cypriniformes, Cobitidae, Botiinae) is a demersal freshwater fish, mainly distributed in various water systems in East and Southeast Asia (Froese and Pauly 2019). It is an important food source in this region. Here, the complete mitochondrial genome sequence of *S. superciliaris* was determined (GenBank accession No. MN709617) and was compared with another *S. superciliaris* mitogenome data reported before. The specimens (Voucher no. 20190528006) were collected from Wulong (29.40 N, 108.05 E), Chongqing, China, and were stored in the museum of the Freshwater Fish Research Laboratory (Zunyi Normal College, Guizhou, China). Primers used in this study and methods for collecting DNA sequences followed the procedures outlined in Wang et al. (2011).

The complete mitochondrial genome of *S. superciliaris* was a circular molecule with 16,572 bp in length. It contained 13 PCGs, 22 tRNA genes, 2 rRNA genes, and one control region, which was consistent with previous results (Ye et al. 2013). Most of the genes were encoded on H-strand, while *ND6* and 8 tRNA genes were encoded on L-strand. The overall nucleotide composition was 31.11% A, 24.81% T, 27.80% C, and 16.27% G, with a slight AT bias. All the mitochondrial PCGs in the *S. superciliaris* use the standard ATG start codon. Three PCGs contain TAA stop codon, three contain TAG stop codon and five contain TGA stop codon, while two contain the incomplete stop codon T––.

Comparing with another mitogenome of *S. superciliaris*, the length of it was 16,597 bp (Accession no. JX683724; Yang et al. 2013). The genetic distance between the two mitogenomes was 0.1%. Twenty variable sites exist in between the two mitogenomes. Among these variable sites, 18 transitions (90%) and 2 transversions (10%) were found. There were 15 variable sites appeared in the PCGs and 4 sites in control region. The other one variable site was occurred in 16S rRNA.

To confirm the phylogenetic position of *S. superciliaris* among Cobitidae, phylogenetic analysis based on the complete mtDNA using maximum-likelihood and Bayesian methods were conducted using IQ-TREE (Nguyen et al. 2015; Hoang et al. 2018) and MrBayes 3.2.6 (Ronquist et al. 2012), respectively. Sequences were aligned using ClustalW algorithm in MEGA7 (Kumar et al. 2016). Since the topology of ML tree and BI tree are highly consistent, only the maximum likelihood tree is shown here (Figure 1). The results from the analyses show that the genus *Sinibotia* is a monophyletic group and *S. superciliaris* demonstrate a sister relationship with *Sinibotia pulchra*.

**Figure 1.**
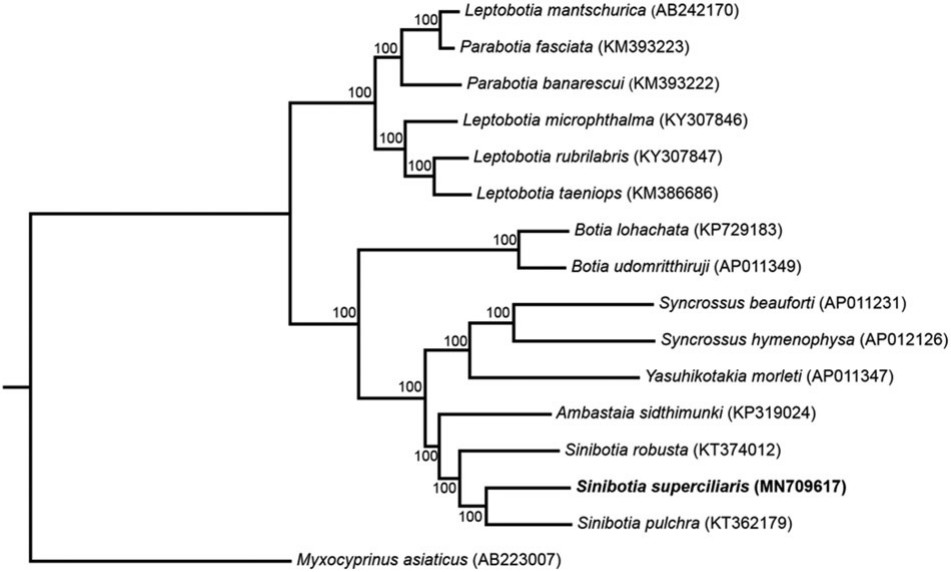
Maximum-likelihood (ML) phylogenetic tree of *Sinibotia superciliaris* and the other 15 species using *Myxocyprinus asiaticus* as an outgroup. The ML bootstrap support values were shown at nodes.
